# Effects of Feedback on Students’ Motor Skill Learning in Physical Education: A Systematic Review

**DOI:** 10.3390/ijerph18126281

**Published:** 2021-06-10

**Authors:** Yulan Zhou, Wei De Shao, Lijuan Wang

**Affiliations:** 1 College of Physical Education and Health Sciences, Zhejiang Normal University, Jinhua 321004, China; zhouyulan004@outlook.com (Y.Z.); txsh@zjnu.cn (W.D.S.); 2 School of Physical Education and Training, Shanghai University of Sport, Shanghai 200438, China

**Keywords:** feedback, motor skill learning, physical education

## Abstract

Objectives: Feedback is an essential factor that may affect students’ motor skill learning during physical education (PE) classes. This review aimed to (1) systematically examine the evidence for the effectiveness of feedback on students’ skill learning during PE classes and (2) summarize the evidence for the effects of feedback elements (i.e., format and content). Methods: A systematic search was conducted on seven electronic databases to identify studies that explored the effects of feedback on student learning during PE classes. Twenty-three studies were selected, and the study quality was evaluated using the Physiotherapy Evidence Database scale. The levels of evidence were determined with the best evidence synthesis. Results: Strong evidence indicates the effectiveness of feedback intervention on students’ skill learning compared with those who received no feedback. Limited evidence was found for the effect of visual feedback compared with verbal feedback. There were mixed results for the effectiveness of information feedback compared with praise or corrective feedback. Conclusion: The current evidence suggests that feedback is useful for skill learning during PE classes. Emergent questions still need to be addressed, such as those regarding the efficiency of using different formats and contents for feedback delivery to enhance motor skill learning during PE classes.

## 1. Introduction

Motor skill competence can be defined as the degree of skilled performance in a wide range of motor tasks as well as the movement quality, coordination, and control underlying a particular motor outcome [1]. Competence in a variety of motor skills (e.g., fundamental skills and athletic skills) is a requisite for promoting physical activity (PA) and physical fitness during childhood and adolescence [2]. Children and adolescents with better motor skill proficiency are more likely to engage in PA than their peers with poorer motor skill competence [3]. The development of motor skills in childhood and adolescence is associated with increased cardiorespiratory fitness, muscular endurance, and muscular strength [4,5]. As most children and adolescents participate in some type of organized physical education (PE) during school, developing and mastering motor skills is one of the core goals of the national PE curriculum [6]. Considering the potential health impact of motor skill competency and the goals of school PE, further investigating the teaching techniques that aid the learning and improvement of motor skills in PE classes is warranted.

Various factors contribute to motor skill learning in PE, such as practice time [7] and teacher optimization [8]. One of the essential factors that may affect motor skill learning is feedback. Feedback has been defined as an action taken by an agent (e.g., teacher and student) to deliver information about one or more aspects of student performance [9,10]. In PE classes, the motor skill learning process focuses on exploring and practicing actions and patterns of movements. Students need information on their motor skill performance to correct errors and improve, as well as to motivate them to continue learning. Therefore, providing regular feedback in the PE setting is vital.

Many studies examined the effects of feedback on students’ motor skill learning in PE lessons and used self-reporting and objective measures (e.g., skill performance assessment manual, measuring tape, and stopwatch) to assess students’ motor skill learning. Several experimental studies confirmed that feedback interventions had a positive impact on students’ motor skill learning [11,12,13]. However, some investigations revealed that feedback has no significant influence on students’ motor skill learning [14,15,16]. The inconsistences among studies suggest that further investigation is needed to investigate the impact of feedback on students’ motor skill learning in PE classes. However, no systematic review has addressed this issue yet. Furthermore, feedback can be delivered through different formats (i.e., verbal, nonverbal, written, and visual/graphical) and content types (i.e., correct–incorrect, normative information, and designed to praise) [17,18]. These elements will generate varied effects on students’ motor skill learning in PE lessons. For example, Niźnikowski et al. (2016) established that visual feedback is more effective in students’ symmetrical movement learning than verbal feedback [19]. Feedback that provides students with specific information may also be more efficient for students’ tossing skills (i.e., an object control skill that is defined as throwing a ball with appropriate force, accuracy, and speed) than praise [20].

Therefore, the purpose of this study was to systematically review the effects of feedback on students’ motor skill learning during PE lessons. Specifically, this research sought to summarize the evidence for (1) the effectiveness of feedback and (2) the effects of feedback elements (i.e., format and content) on students’ skill learning during PE classes. The findings of this review may provide simple and clear feedback guidance for PE teachers, such that feedback elements, which are known to be the most effective features for promoting student motor skill learning, are incorporated.

## 2. Methods

This review adhered to the preferred reporting items for systematic reviews and meta-analysis statement [21].

### 2.1. Search Strategy 

A systematic search of the relevant literature published until April 2021 was conducted in seven electronic databases, namely: Academic Search Premier, ERIC, MEDLINE, the Psychology and Behavioral Sciences Collection, Scopus, SPORTDiscus, and Web of Science. The search strategy included the following combinations of variations of keywords: (1) feedback OR comment* OR response OR evaluat* OR assess*; (2) physical education OR PE OR lesson* OR class* OR curricul* OR school* OR instruct* OR student*; and (3) motor skill* OR motor competen* OR motor development OR motor performan* OR motor abilit* OR motor function* OR motor proficiency OR motor learning OR movement skill* OR movement abilit* OR movement proficiency OR actual competen* OR skill proficiency OR athletic skill* OR sport skill* OR fundamental movement OR basic movement OR gross motor OR motor coordination OR locomotor skill* OR object control OR fine motor OR manipulative skill OR object manipulation. The search strategies used for each database are shown in the Supplementary Materials (Table S1). A manual search was also conducted among the reference lists of all included studies to identify additional relevant papers.

### 2.2. Inclusion and Exclusion Criteria

The inclusion and exclusion criteria for this review were as follows. (1) Studies must be a deliberate attempt to implement a change to the usual feedback so as to promote motor skill learning. Cross-sectional and cohort designs were disregarded. (2) Articles delivered in school PE classes were included; those focusing on other settings were excluded. (3) Studies on general elementary school students or secondary school students were covered. Pre-school, post-secondary institution, or disability samples were excluded. (4) Published peer-reviewed journals with full-text articles in English until April 2021 were included, thereby excluding unpublished articles, conference proceedings, dissertations, theses, and reviews. Two reviewers independently assessed the eligibility of the studies according to these inclusion and exclusion criteria. Any disagreements between the two reviewers were resolved by a third one.

### 2.3. Data Extraction

Data were extracted from each study by using a common table form that includes the following information: name of first author, year of publication, geographic location, participant characteristics, study design, task complexity, feedback elements (e.g., feedback provider, feedback format, and feedback content), dependent variables, and results. The tasks were categorized into simple and complex tasks according to the movement required (discrete, continuous, and serial skills) and the perceptual attributes involved (open or closed) [22]. 

### 2.4. Quality Assessment

The methodological quality of the studies was evaluated using the Physiotherapy Evidence Database (PEDro) scale, which is used to assess the methodological quality of studies in similar areas [23,24]. The PEDro scale contains 10 items that evaluate: aspects of randomization (two items), the blinding technique (three items), data reporting (three items), data analysis (one item), and adequacy of follow-up (one item). Each item was scored as 1 (the assessed item was explicitly present) or 0 (the assessed item was absent). The final score was obtained by adding the score obtained across relevant items and ranges from 0 to 10. High-quality studies have scores of 5 or higher, and low-quality counterparts have scores under 5 [25]. Two reviewers independently assessed the methodological quality of the included studies. In the case of a disagreement, a consensus was reached through discussion with the third reviewer.

### 2.5. Data Syntheses and Analysis 

Given that a wide variety of feedback elements were used in the included studies, a meta-analysis was not conducted. Instead, a summary of the results was measured using the best evidence synthesis [26]. This rating system considers the number, methodological quality, and consistency of the outcomes of the studies using five levels of evidence: (1) strong evidence, provided by generally consistent findings in multiple (≥3) high-quality studies; (2) moderate evidence, provided by generally consistent findings in two high-quality studies; (3) limited evidence, provided by generally consistent findings in one high-quality study; (4) conflicting evidence, provided by contradictory findings in studies; and (5) insufficient/no evidence, provided by generally consistent findings in only low-quality studies [27]. Consistency of outcomes was characterized by significant results in the same direction reported in at least two-thirds of the relevant studies [26].

## 3. Results

### 3.1. Study Selection

**Figure 1:**
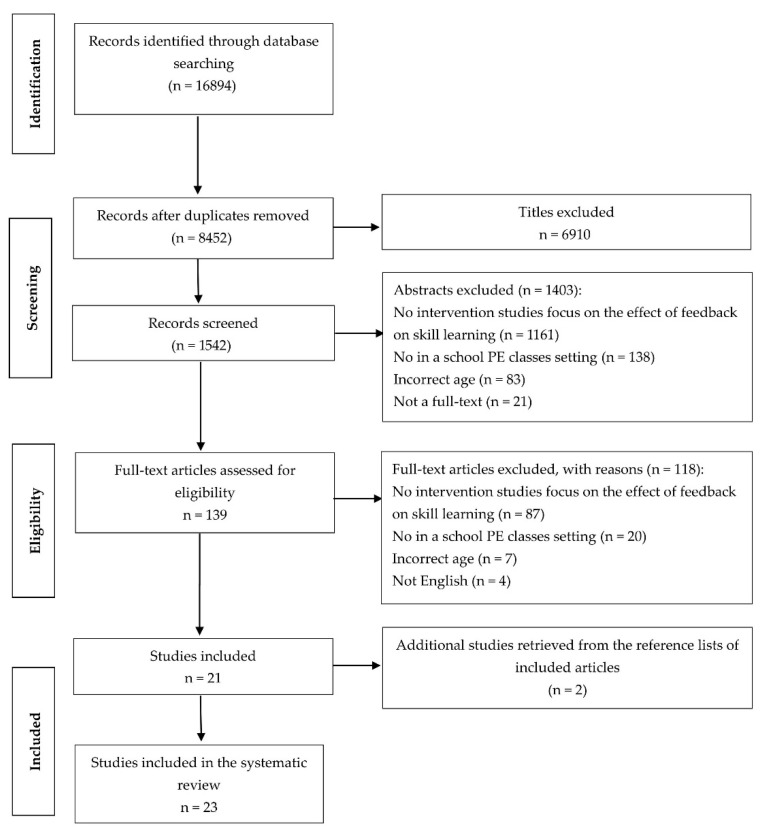
Flow diagram of the study selection process.

A total of 16,894 studies were identified through a search of the initial databases. After removing duplicates and reviewing titles and abstracts, 139 studies were identified as potentially relevant. Of these, 21 studies met the inclusion and exclusion criteria. The search of reference lists from relevant studies and reviews yielded two additional studies. Therefore, the final number of studies identified in the review was 23. Figure 1 summarizes the process of the literature research.

### 3.2. Study Description

Table 1 presents the characteristics of the included studies in this review. The publication period was from 1987 to 2019, with five studies from before 2000, five studies from 2000 to 2009, and 13 studies from 2010 to 2019. The investigations were primarily conducted in the United States of America (10 studies); two studies each from France, Greece, and Australia; and one each from the United Kingdom, Germany, the Netherlands, Poland, Spain, Athens, and Ireland. Relatively large variability in sample sizes was observed across studies. Specifically, the sample ranged from 3 to 950, with 16 studies having less than 100 subjects and only seven studies having over 100 subjects. Among the reviewed studies, 12 were conducted in elementary schools (i.e., Grades 1–6; approximately 5–12 years old), seven in middle schools (i.e., Grades 6–8; approximately 12–14 years old), and the remaining four were conducted in high school settings (i.e., Grades 9–12; approximately 14–18 years old). Moreover, five studies were randomized clinical trials (RCTs), two were quasi-experimental studies, six were control experiment trials, and 10 were pre-post designed studies. Fourteen of the 23 studies assigned simple tasks, seven studies explored complex tasks, and the remaining two studies included simple and complex task conditions. In all included studies, teachers provided feedback across eight studies, peers provided feedback in four, and feedback was provided by video in two studies. Teachers, peers, videos, and instructors provided feedback in nine studies.

### 3.3. Methodological Quality

The methodological quality of the 23 included studies is shown in Table 2. The PEDro scores of these studies ranged from 1 to 7. According to the assessment scale, eight studies had scores of 5 or higher and were thus considered to be of high quality. Fifteen studies obtained scores below 5 and were categorized as low quality. The main area of methodological weakness among the included studies was related to the blinding technique—that is, the possible score of the combined individual scores of participants, intervention providers, and evaluator blinding. Only one study obtained a score of 2; four studies scored 1 for the blinding technique, and the majority of the studies (18 studies) received a score of 0.

### 3.4. Summary of the Feedback Outcome

Studies included in this review used different feedback elements for the intervention design. We aimed to provide a comprehensive evaluation of the evidence and did not impose any restriction on the type of comparisons used in these studies. Therefore, these experimental studies were divided into three groups of comparison: with versus without feedback, visual versus verbal feedback, and information feedback versus praise or corrective feedback. A study with more than one category of comparison or result was considered as several distinct studies in all subsequent analyses. The comparisons that were reported in less than three studies were not summarized as evidence.

#### 3.4.1. Comparison 1: The Effect of the Presence of Feedback Versus the Absence of Feedback

Thirteen studies investigated the effect of the presence versus the absence of feedback on students’ motor skill learning. Ten studies found that feedback can significantly improve students’ basketball [11], volleyball [30,36], striking [33], standing broad jump [28], soccer [12], table tennis skills [13], gymnastics [39], cup-stacking [34], and fundamental motor skills [37] compared to counterparts who failed to receive any feedback. However, the remaining three studies indicated no significant differences in students’ volleyball [29], cup-stacking [34], and lacrosse skills [15] for experimental groups receiving feedback intervention versus control groups that received no feedback. Out of 13 studies, 10 studies (76.9%, including three high-quality studies [12,28,34]) reported the effectiveness of feedback on students’ motor skill learning. According to the best-evidence synthesis, strong evidence indicates that feedback can improve students’ motor skill learning compared with receiving no feedback.

#### 3.4.2. Comparison 2: The Effect of Visual Versus Verbal Feedback

Eight studies compared the effects of visual and verbal feedback on students’ motor skill learning. Three studies compared verbal and visual feedback used alone, and all revealed the significant effect of visual feedback on students’ gymnastic skills [42], symmetrical movement skills [19], and front crawl skills [41]. Three studies compared students’ volleyball skills between expert-model demonstration and verbal feedback and self-model and verbal feedback. Two studies showed significant improvement of the skill in the expert-model and verbal feedback group [32,35]. The remaining study found no significant differences [16]. Palao et al. (2015) investigated the effectiveness of teacher verbal feedback, visual and teacher verbal feedback, and visual and student verbal feedback on students’ hurdle-skill learning [40]. The visual and teacher verbal feedback group showed the highest hurdle skills. Kok et al. (2019) compared students’ shot-put skills among those who received self-controlled visual and verbal feedback, externally controlled visual and verbal feedback, and verbal feedback [14]. The results showed no significant differences in skill improvements among the groups. In this group comparison, visual feedback was compared with verbal feedback, and expert-model and verbal feedback was compared with self-model and verbal feedback in three or more studies. Three studies (100%, including one high-quality study [42]) and two studies (66.7%, including one high-quality study [16]) reported that students performed higher motor skill scores when they received visual feedback and expert-model and verbal feedback, respectively. Therefore, limited evidence exists for the effects of these types of feedback.

#### 3.4.3. Comparison 3: The Effect of Information Feedback Versus Praise or Corrective Feedback

Ten studies involved information feedback compared with praise or corrective feedback. Four of seven studies confirmed that providing individual information feedback has more beneficial effects on students’ volleyball [35], tossing [20], cup-stacking [34], and skill practice [31] than praise or corrective feedback. By contrast, three studies found no significant differences in students’ lacrosse [15], volleyball [16], and cup-stacking skills [34]. The remaining three studies compared the effectiveness of students’ motor skill learning between praise and information feedback, and simple praise. Two studies reported that the combination of praise and information feedback benefits students’ cup-stacking [34] and overhand throw skill learning [38] more than using praise alone, but the other study found no significant differences in students’ cup-stacking skills [34]. Compared with praise or corrective feedback, conflicting evidence was found for the effect of information feedback (57.1%, 4/7 of the studies) on student motor skill learning. Consistent findings (66.7%, 2/3 of the studies, including two high-quality studies [34,38]) reported the increased effectiveness of the combination of praise and information feedback on students’ motor skill learning compared to praise alone. According to the best-evidence synthesis, moderate evidence was found for this type of comparison. 

## 4. Discussion

The purpose of the present systematic review was to provide a synthesis of the current evidence regarding the effect of feedback on students’ motor skill learning during PE lessons. The final analysis included 23 studies conducted in 11 countries. Given that more than half of studies (56.5%) were published after 2009, we speculate that scholars worldwide have become more concerned about feedback within the last decade, with the well-documented importance of feedback on student motor skill learning during PE classes.

### 4.1. Comparison 1: The Effect of the Presence versus the Absence of Feedback

In this systematic review, 13 studies compared the presence versus the absence of feedback intervention. The results and methodological qualities of the studies reveal strong evidence for the effectiveness of obtaining feedback on students’ skill learning compared with receiving no feedback. This result aligns with previous meta-analyses that have reported the significant effect of feedback on student learning [43]. The influence of feedback on students’ motor skill learning during PE classes could be explained by two reasons. First, the feedback (e.g., comments, suggestions, and directions) delivered in response to students’ specific actions or performance can provide information about the techniques and enable them to identify their technical errors, which constitute an important aspect for skill learning [40]. Second, feedback may promote a positive student–teacher relationship, a feature that has been shown to increase students’ skill learning motivation, thereby improving their skill performance [44]. Providing feedback may be a natural, efficient, and economical strategy for students’ motor skill learning during PE classes. Therefore, the teachers should regularly use this teaching strategy.

### 4.2. Comparison 2: The Effect of Visual versus Verbal Feedback

In the current review, the format of delivering the feedback varied with the verbal and visual modes of feedback used. Teacher verbal feedback, defined as anything a teacher says that provides students with some type of information, was the most common format of feedback identified in pedagogical research in the PE setting [45]. Visual feedback used during PE lessons involves video recordings to interpret and analyze students’ objective performance in their process of executing tasks [46]. In the process of skill learning, the performance of an individual is fleeting, and the information cannot be detected by their sensory system [47]. Visual feedback can provide students’ static and dynamic images in action and give them a clear idea of the executions that they have completed or those they are about to attempt [48]. Notwithstanding being supported by limited evidence, students seem to improve their motor skills to a higher degree from visual feedback compared with verbal feedback. In addition, limited evidence was found for the effect of the expert-model (i.e., video demonstration of the skill of elite athletes) and verbal feedback on students’ motor skill learning than the self-model (i.e., video demonstration of the skill by the students) and verbal feedback. The perfect execution of skills by an expert seemed to motivate students to imitate and strive more toward a better performance of the skill [16]. Furthermore, students who received the expert-model demonstration were able to focus more attention on their execution of the skill. By contrast, students who received a self-model needed to receive information on correcting errors and improving performance. More than one source of information could decrease the likelihood of a perfect execution [49]. Therefore, the expert-model group performed better skills than the self-model group. 

Of the present comparison, Palao et al. (2015) reported that visual and teacher verbal feedback provided significant effects on student skill learning, compared to visual and student verbal feedback [40]. Given the higher level of content and pedagogical content knowledge that would be expected of a teacher relative to students, this result is unsurprising. However, teachers are faced with numerous PE students and they cannot provide enough feedback to each one [50]. Having students provide feedback to peers or on peers’ performance of a task as an educationally appropriate strategy to enhance student learning will become increasingly important. Kok et al. (2019) examined self-controlled visual feedback (i.e., students decide after every trial whether they want to obtain feedback) and externally-controlled visual feedback (i.e., the experimenter informs students after every trial whether or not they will obtain feedback) [14,51]. As expected, differences in feedback frequency were observed between self-controlled and externally-controlled groups. Few studies explored the effect of feedback frequency on student skill learning during PE classes. Moreover, prior researchers suggested that students in the early stages of learning benefited more from higher relative frequencies of feedback than their more experienced counterparts [52]. The acquisition of simpler skills benefited more from reduced relative frequencies of feedback than that for more complex skills [53,54]. Future research should be designed as intervention studies to address this issue.

### 4.3. Comparison 3: The Effect of Information Feedback versus Praise or Corrective Feedback

For the third group of comparison, the agents (e.g., teachers and students) provided a wide range of feedback content, including praise (i.e., statements such as “good”, “great”, “excellent”, and “wonderful” or nonverbal feedback such as smile, high fives, pat, and applause, which are intended to praise); information feedback (i.e., the information related to specific movement characteristics); and corrective feedback (i.e., teachers or peers’ reactions to some errors in the learning process or doing tasks from the students’ end). Information feedback consciously reinforces students’ explicit or declarative memory, and their acquisition can occur quickly in this learning process [55]. Praise may be helpful to create a positive, supportive learning environment for students and enhance motivation but may not consistently improve students’ skill learning compared with information feedback [56]. A commonly accepted situation within the PE teaching and research community is that the combination of praise and information feedback may have more benefits for students’ skill learning than praise alone [20,34,57]. 

Present review reported conflicting evidence for the effect of information feedback used alone compared with that of praise or corrective feedback. This finding is expected given that the effect of feedback content on student motor skill learning during PE classes is moderated by numerous factors, such as the task complexity and students’ initial ability. Previous studies indicated that for complex skills, the combination of corrective feedback and praise seem to be effective because it is perceived by the students as supportive information that leads to self-confidence improvement [58]. In the case of simple skills, information feedback on some form of deficiency that students needed to pay closer attention to is enough [15]. Furthermore, the effectiveness of different feedback content on students’ motor skill learning depended on their initial ability. Fitts and Posner proposed three stages of learning: the cognitive, associative, and autonomous stages [59]. Beginners are in the cognitive stage of learning, and it was crucial for them to pay attention to the instructions and demands of the motor skill task to be learned. Thus, information feedback is better for beginners who need to know how to execute the skill movement [60]. Experienced students are in the autonomous stage of learning, which is considered a relatively automatic stage wherein students are able to correct their errors [61]. Therefore, students given corrective feedback about the directions of error (faster or slower, or more or less) could lead to an increase in skill accuracy and hasten skill learning [52]. Given the potential factors that may influence the effectiveness of feedback on students’ learning, summarizing consistent results about which type of feedback content is suitable for students’ motor skill leaning is challenging. Future studies should generate evidence on how the complexity of the task and student background influence the effectiveness of feedback on students’ motor skill learning during PE classes.

### 4.4. Methodological Quality of the Studies

Most of the reviewed studies were categorized as having low quality (65.2%). Data on quality assessment suggest that the weak points of the studies are mainly related to the blinding technique. None of the analyzed studies blinded evaluators; in only one study the therapist was blinded, and in only five studies the participants were blinded. Although blinding participants, therapists, and evaluators in education research is difficult, the low PEDro scale scores of the analyzed studies suggest their weakness in terms of experimental methodology. Therefore, a generalized conclusion must be regarded with caution, and further research based on high-quality study designs are needed to strengthen the evidence of this systematic review.

## 5. Limitations and Directions for Future Research

Some limitations inherent within the review should be noted. First, despite the thorough literature search, a few published studies were possibly overlooked because of the keywords that may differ from those used in the current work. Second, most studies had small sample sizes (69.6%, 16 studies), lacked randomization (78.3%, 18 studies), and were of low quality (65.2%, 15 studies), thereby limiting the generalizability of the findings and the summarization of strong evidence. Finally, the feedback contexts and comparison among different feedback contents and feedback formats were not the same across the involved studies. Therefore, achieving a deeper understanding of the outcomes of these groups of comparisons was difficult.

Despite the outlined limitations, the review highlights several areas of future research related to feedback on students’ skill learning during PE classes. To expand upon the current study, further research can explore high-quality RCTs to provide conclusive evidence for the effects of feedback. In addition, the use of computers and other technologies gives unique opportunities for teachers to provide visual feedback to students [62]. Subsequent research must explore how to improve the efficiency of video feedback. Future studies should also delineate the feedback content, the context in which the skill is being taught, and the students’ background to allow researchers and teachers to fully understand the intervention and its intended effects [54]. Given the dearth of research on feedback frequency, additional studies in this area should be designed to further understand this element.

## 6. Conclusions

We systematically collated the available evidence regarding the impacts of feedback on students’ skill learning during PE classes and found strong evidence for the usefulness of feedback on student skill learning during those classes. Limited evidence suggests a stronger effect of visual feedback compared to verbal feedback. Conflicting evidence was reported for the effectiveness of information feedback compared with praise or corrective feedback. Moderate evidence was found for the effect of the combination of praise and information feedback on students’ motor skill learning compared to that of praise alone. Additional studies that involve more participants and are methodologically better designed and executed are needed to identify the optimal combinations of feedback elements that are most effective in helping students’ motor skill learning across different contexts.

## Figures and Tables

**Table 1:** Study characteristics of included studies.

First Author (Year)	Geographic Location	Sample Characteristics	Research Design	Task Complexity	Feedback Provider	Feedback Format	Feedback Content	Dependent Variables	Results
Masser (1987) [28]	USA	529 elementary school students, K6	Quasi-experiment	Simple	Teacher	Group 1: no Group 2: verbal	Group 1: no Group 2: praise and information	Standing broad jump	Group 2 reached a higher mean score
Sariscsany (1995) [29]	USA	3 middle school students K6–7	Control experiment	Simple	Teacher	Group 1: no Group 2: verbal Group 3: verbal	Group1: no feedback Group 2: information Group 3: information	Volleyball	No differences among groups
Pellett (1995) [30]	USA	68 middle school students K7–8	Pre-post	Simple & complex	Teacher	Group 1: no Group 2: verbal	Group 1: no Group 2: information and corrective	Volleyball	Group 2 significantly improved students’ skills
Lounsbery (1999) [31]	USA	950 middle school students K7–9	Pre-post	Simple	Teacher	Verbal	Group 1: corrective Group 2: information	Soccer, hockey, volleyball, etc.	Group 2 positively connected to students’ appropriate skill practice
Zetou (1999) [32]	Greece	58 elementary school students, 11.7 years old	RCT	Complex	Video and teacher	Group 1: self-model and verbal Group 2: expert-model and verbal	information	Volleyball skill	Group 2 significantly improved the skill
Johnson (2001) [33]	USA	56 elementary school students K3	Pre-post	Complex	Student	Group 1: no Group 2: verbal	Group 1: no Group 2: corrective	Striking	Group 2 had a higher percentage of correct trials
Fredenburg (2001) [34]	USA	103 elementary school students K4	RCT	Simple & complex	Teacher	Verbal	Group 1: no Group 2: praise Group 3: information Group 4: praise and information	Cup-stacking	No significant differences in simple tasks Group 3 and Group 4 had higher performance scores in complex tasks
Zetou (2002) [35]	Greece	116 elementary school students, 11.7 years old	RCT	Simple	Video, teacher, and instructor	Group 1: self-model and verbal Group 2: expert-model and verbal	Group 1: corrective; Group 2: information	Volleyball skill	Group 2 significantly improved the skill
Barzouka (2007) [16]	Athens	53 high school students, 13.1 years old	RCT	Complex	Video and teacher	Group 1: expert-model and verbal Group 2: self-model and verbal Group 3: verbal	Group 1: information Group 2: corrective Group 3: corrective	Volleyball	No significant differences
Ayvazo (2009) [36]	USA	4 middle school students K6	Pre-post	Simple	Student	Group 1: no Group 2: verbal	Group 1: no Group 2: information	Volleyball	Group 2 improved performance
Ensergueix (2010) [13]	France	72 high school students, 15.1 years old	Control experiment	Simple	Student	Group 1: no Group 2: verbal	Group 1: no Group 2: praise and corrective	Table tennis	Group 2 had a higher score
Casey (2011) [37]	Australia	27 elementary school students, 7 years old	pre-post	Simple	Video	Group 1: no Group 2: visual	Group 1: no Group 2: information	Fundamental Motor Skills	Group 2 had a higher motor skill performance
Cohen (2012) [38]	USA	97 elementary school students, 8.8 years old	Control experiment	Simple	Teacher	Verbal	Group 1: praise Group 2: praise and information	Overhand throw	Group 2 enhanced students’ performances
Brooker (2013) [39]	UK	30 elementary school students K2	pre-post	Simple	Video	Group 1: no Group 2: visual	Group 1: no Group 2: information	Gymnastic	Group 2 improved children’s technique
O’Loughlin (2013) [11]	Ireland	23 elementary school students, 9–10 years old	Pre-post	Simple	Video and teacher	Group 1: no Group 2: visual	Group 1: no Group 2: information	Basketball	Group 2 improved student performance
Palao (2015) [40]	Spain	60 high school students, 15 years old	Pre-post	Complex	Video, teacher, and students	Group 1: teacher verbal Group 2: visual and teacher verbal Group 3: visual and student verbal	corrective	Hurdle skills	Group 2 provided the most positive overall results
Drost (2015) [20]	USA	170 elementary school students 10.6 years old	Pre-post	Simple	Teacher	Verbal	Group 1: praise Group 2: information	Tossing skill	Group 2 resulted in an improved skill performance
Whipp (2015) [12]	Australia	106 high school students 12.5 years old	Control experiment	Simple	Students	Verbal	Group 1: no Group 2: information and corrective	Soccer	Group 2 improve student performance
Niźnikowski (2016) [19]	Poland	13 elementary school students 7–8 years old	Pre-post	Complex	Video and teacher	Group 1: verbal Group 2: visual	information	Symmetrical movement	Group 2 achieved higher performance
Drost (2017) [15]	USA	113 elementary school students 10–11 years old	Quasi-experiment	Complex	Teacher	Verbal	Group 1: no Group 2: praise Group 3: information	Lacrosse	No significant differences
Kretschmann (2017) [41]	Germany	31 middle school K5	RCT	Complex	Video and teacher	Group 1: verbal Group 2: visual	information	Front crawl skill	Group 2 significantly improve the skill
Potdevin (2018) [42]	France	43 middle school students 12.4 years old	Control experiment	Simple	Video and teacher	Group 1: verbal Group 2: visual	information	Gymnastic	Group 2 had better skill performance
Kok (2019) [14]	Netherlands	56 middle school students 12.7 years old	Control experiment	Simple	Video and teacher	Group 1: externally controlled video and verbal Group 2: self-controlled video and verbal Group 3: verbal feedback	information	Shot-put	No significant differences

**Table 2:** PEDro Scores.

Reference	Eligibility Criteria	Random Allocation	Concealed Allocation	Groups Similar at Baseline	Participants Blinded	Provider Blinded	Evaluator Blinded	Follow-Up	Intention-to-Treat Analysis	Between-Group Comparison	PEDro Score
Masser (1987) [28]	1	1	0	1	0	0	0	1	0	1	5
Sariscsany (1995) [29]	0	0	0	0	0	0	0	1	0	1	2
Pellett (1995) [30]	0	0	0	0	0	0	0	1	0	1	2
Lounsbery (1999) [31]	0	0	0	1	0	0	0	0	0	1	2
Zetou (1999) [32]	0	1	0	0	0	0	0	1	0	1	3
Johnson (2001) [33]	0	0	0	0	0	0	0	1	0	1	2
Fredenburg (2001) [34]	0	1	1	0	1	1	0	1	1	1	7
Zetou (2002) [35]	0	1	0	0	0	0	0	1	0	1	3
Barzouka (2007) [16]	1	1	1	1	0	0	0	1	1	1	7
Ayvazo (2009) [36]	0	0	0	0	0	0	0	1	0	0	1
Ensergueix (2010) [13]	1	0	0	1	0	0	0	1	0	1	4
Casey (2011) [37]	0	0	0	1	0	0	0	0	0	0	1
Cohen (2012) [38]	1	1	0	1	0	0	0	1	0	1	5
Brooker (2013) [39]	0	0	0	0	0	0	0	1	0	0	1
O’Loughlin (2013) [11]	0	0	0	0	0	0	0	1	0	0	1
Palao (2015) [40]	0	0	0	1	1	0	0	0	0	1	3
Drost (2015) [20]	0	1	0	1	0	0	0	0	0	1	3
Whipp (2015) [12]	0	1	1	1	1	0	0	0	0	1	5
Niźnikowski (2016) [19]	0	1	0	1	0	0	0	1	0	1	4
Drost (2017) [15]	1	1	0	1	0	0	0	1	0	1	5
Kretschmann (2017) [41]	0	1	0	0	0	0	0	1	0	1	3
Potdevin (2018) [42]	1	0	0	1	1	0	0	1	0	1	5
Kok (2019) [14]	1	0	1	1	1	0	0	1	0	1	6

## Data Availability

Not applicable.

## Supplementary Materials

The following are available online at https://www.mdpi.com/article/10.3390/ijerph18126281/s1, Table S1: Search strategies.
