# Supplementary material for: Effects of Feedback on Students’ Motor Skill Learning in Physical Education: A Systematic Review
Source: Int J Environ Res Public Health. 2021 Jun 10;18(12):6281. doi: 10.3390/ijerph18126281 (PMC8296044; doi:10.3390/ijerph18126281)
Supplement: Supplementary file 1 [file ijerph-18-06281-s001.zip › ijerph-1252532-supplementary.pdf]

Table S1. Search strategies

**Database: Academic Search Premier**<br>**Number of results: 3972**<br>((feedback OR comment* OR response OR evaluat* OR assess*) AND (physical education OR PE OR lesson* OR class* OR curricul* OR school* OR instruct* OR student*) AND (motor skill* OR motor competen* OR motor development OR motor performan* OR motor abilit* OR motor function* OR motor proficiency OR motor learning OR movement skill* OR movement abilit* OR movement proficiency OR actual competen* OR skill proficiency OR athletic skill* OR sport skill* OR fundamental movement OR basic movement OR gross motor OR motor coordination OR locomotor skill* OR object control OR fine motor OR manipulative skill OR object manipulation)) in Title, Abstract, Keywords.<br>Filters: English
**Database: ERIC**<br>**Number of results: 1725**<br>((feedback OR comment* OR response OR evaluat* OR assess*) AND (physical education OR PE OR lesson* OR class* OR curricul* OR school* OR instruct* OR student*) AND (motor skill* OR motor competen* OR motor development OR motor performan* OR motor abilit* OR motor function* OR motor proficiency OR motor learning OR movement skill* OR movement abilit* OR movement proficiency OR actual competen* OR skill proficiency OR athletic skill* OR sport skill* OR fundamental movement OR basic movement OR gross motor OR motor coordination OR locomotor skill* OR object control OR fine motor OR manipulative skill OR object manipulation)) in Title, Abstract, Keywords.<br>Filters: English
**Database: MEDLINE**<br>**Number of results: 889**<br>((feedback OR comment* OR response OR evaluat* OR assess*) AND (physical education OR PE OR lesson* OR class* OR curricul* OR school* OR instruct* OR student*) AND (motor skill* OR motor competen* OR motor development OR motor performan* OR motor abilit* OR motor function* OR motor proficiency OR motor learning OR movement skill* OR movement abilit* OR movement proficiency OR actual competen* OR skill proficiency OR athletic skill* OR sport skill* OR fundamental movement OR basic movement OR gross motor OR motor coordination OR locomotor skill* OR object control OR fine motor OR manipulative skill OR object manipulation)) in Title, Abstract, Keywords.<br>Filters: English
**Database: Psychology and Behavioral Sciences Collection**<br>**Number of results: 1914**<br>((feedback OR comment* OR response OR evaluat* OR assess*) AND (physical education OR PE OR lesson* OR class* OR curricul* OR school* OR instruct* OR student*) AND (motor skill* OR motor competen* OR motor development OR motor performan* OR motor abilit* OR motor function* OR motor proficiency OR motor learning OR movement skill* OR movement abilit* OR movement proficiency OR actual competen* OR skill proficiency OR athletic skill* OR sport skill* OR fundamental movement OR basic movement OR gross motor OR motor coordination OR locomotor skill* OR object control OR fine motor OR manipulative skill OR object manipulation)) in Title, Abstract, Keywords.<br>Filters: English

**Database: Scopus**<br>**Number of results: 5828**<br>TITLE-ABS-KEY (feedback OR comment* OR response OR evaluat* OR assess*) AND (physical education OR PE OR lesson* OR class* OR curricul* OR school* OR instruct* OR student*) AND (motor skill* OR motor competen* OR motor development OR motor performan* OR motor abilit* OR motor function* OR motor proficiency OR motor learning OR movement skill* OR movement abilit* OR movement proficiency OR actual competen* OR skill proficiency OR athletic skill* OR sport skill* OR fundamental movement OR basic movement OR gross motor OR motor coordination OR locomotor skill* OR object control OR fine motor OR manipulative skill OR object manipulation)<br>Filters: English
**Database: SPORTDISCUS**<br>**Number of results: 2374**<br>((feedback OR comment* OR response OR evaluat* OR assess*) AND (physical education OR PE OR lesson* OR class* OR curricul* OR school* OR instruct* OR student*) AND (motor skill* OR motor competen* OR motor development OR motor performan* OR motor abilit* OR motor function* OR motor proficiency OR motor learning OR movement skill* OR movement abilit* OR movement proficiency OR actual competen* OR skill proficiency OR athletic skill* OR sport skill* OR fundamental movement OR basic movement OR gross motor OR motor coordination OR locomotor skill* OR object control OR fine motor OR manipulative skill OR object manipulation)) in Title, Abstract, Keywords.<br>Filters: English
**Database: Web of Science**<br>**Number of results: 192**<br>((feedback OR comment* OR response OR evaluat* OR assess*) AND (physical education OR PE OR lesson* OR class* OR curricul* OR school* OR instruct* OR student*) AND (motor skill* OR motor competen* OR motor development OR motor performan* OR motor abilit* OR motor function* OR motor proficiency OR motor learning OR movement skill* OR movement abilit* OR movement proficiency OR actual competen* OR skill proficiency OR athletic skill* OR sport skill* OR fundamental movement OR basic movement OR gross motor OR motor coordination OR locomotor skill* OR object control OR fine motor OR manipulative skill OR object manipulation)) in Title, Abstract, Keywords.<br>Filters: English
